# Implicit Semantic Processing of Linguistic and Non-linguistic Stimuli in Adults with Autism Spectrum Disorder

**DOI:** 10.1007/s10803-020-04736-5

**Published:** 2021-02-05

**Authors:** Emme O’Rourke, Emily L. Coderre

**Affiliations:** 1 Department of Communication Sciences and Disorders, University of Vermont, 489 Main St., Burlington, VT 05405 USA; 2 Present Address: St. Albans City School in the Maple Run Unified School District, St. Albans, VT USA

**Keywords:** Semantic integration, Autism, Event-related potentials, N400

## Abstract

While many individuals with autism spectrum disorder (ASD) experience difficulties with language processing, non-linguistic semantic processing may be intact. We examined neural responses to an implicit semantic priming task by comparing N400 responses—an event-related potential related to semantic processing—in response to semantically related or unrelated pairs of words or pictures. Adults with ASD showed larger N400 responses than typically developing adults for pictures, but no group differences occurred for words. However, we also observed complex modulations of N400 amplitude by age and by level of autistic traits. These results offer important implications for how groups are delineated and compared in autism research.

The rate of diagnosis for autism spectrum disorders (ASD) has increased over the last decade, with the most recent estimate to be one in every 54 children with the diagnosis (Center for Disease Control and Prevention 2020). This group of neurodevelopmental disorders is characterized by deficits in social communication and restricted and repetitive behaviors (American Psychiatric Association 2013). Although the severity of autistic traits can vary between individuals both with and without an ASD diagnosis, many individuals with ASD experience difficulties with language, specifically in higher-level functions such as semantic processing: understanding of the meaning of a stimulus, regardless of the mode of presentation (e.g., word, picture, or sound) (Coderre et al. 2017). Difficulties with language processing in individuals with ASD may be particularly tied, at least in part, to difficulties with *semantic integration*, which involves not just accessing the semantics of stimuli but assimilating meanings together to arrive at a holistic understanding or a “gist”.

One way of quantifying semantic integration is through event related potentials (ERPs), which are derived from the electroencephalogram (EEG). In previous research with typically developing (TD) populations, the N400 component of the ERP, a negative-going deflection peaking approximately 400 ms after stimulus presentation, has been linked to semantic processing and integration (Kutas and Hillyard 1980; Lau et al. 2008), although a number of other functional interpretations exist (e.g., Brouwer et al. 2012; see Kutas and Federmeier 2011 for a broader discussion). The amplitude of the N400 component is typically reduced when a sentence-final word is semantically congruent with the preceding context of the sentence. Consider the sentences, “the bird has yellow *feathers*” and “the bird has yellow *shoes*.” The word ‘*feathers’* is semantically congruent with the beginning of the sentence, while ‘*shoes’* is incongruent. The difference in N400 amplitude between the congruent and incongruent conditions here is termed the “N400 effect.” The same modulation of N400 amplitude occurs for single words when the two words are related (e.g., bird-FEATHERS) compared to when they are unrelated (e.g., bird-SHOES).

Several studies have reported that individuals with ASD show reduced or absent N400 effects for language compared to TD individuals, suggesting impairments in semantic integration (Braeutigam et al. 2008; Dunn et al. 1999; Dunn and Bates 2005; McCleery et al. 2010; Pijnacker et al. 2010; Strandburg et al. 1993; Verbaten et al. 1991). These findings have been explained in light of the theory of weak central coherence (Frith 1989), which proposes that individuals with ASD have a tendency to focus on details at the expense of the broader picture. Therefore difficulties with language processing in ASD may arise from a reduced use of semantic context to process the meanings of words. Difficulties with semantic integration have been documented for pairs of single words (e.g. Dunn et al. 1999; McCleery et al. 2010) and are likely compounded with increasing complexity such as in sentences (e.g. Braeutigam et al. 2008; Pijnacker et al. 2010) or narratives (Coderre et al. 2018).

In contrast to these difficulties with semantic integration of language, there have also been reports that semantic integration of non-verbal stimuli like pictures or sounds is intact in individuals with ASD (Kamio and Toichi 2000; McCleery et al. 2010), which might suggest a language-specific difficulty with semantic integration. However, these previous studies used cross-modal stimuli (i.e. combinations of words and pictures), making it difficult to determine whether a true language-specific difficulty with semantic processing exists in this population. In previous work, we performed the first within-modality test of semantic integration in linguistic and non-linguistic modalities (Coderre et al. 2017). In a semantic priming ERP paradigm, adults with ASD and TD adults were shown pairs of semantically related or unrelated words or pictures. As reported by prior literature, there were no differences in N400 effect magnitudes (i.e. amplitude differences between related and unrelated pairs) for picture stimuli, suggesting intact semantic processing of non-linguistic information in individuals with ASD. Surprisingly, both groups also showed similar magnitudes of N400 effects in response to word stimuli, which contradicts previous reports of impaired semantic processing of linguistic stimuli in individuals with ASD (Braeutigam et al. 2008; Dunn and Bates 2005; Dunn et al. 1999; McCleery et al. 2010; Pijnacker et al. 2010; Strandburg et al. 1993; Verbaten et al. 1991). We speculated in this previous study that this surprising result could have arisen from the use of an explicit task: participants were asked to judge whether each pair of pictures or words was related or unrelated. Although individuals with ASD are thought to have a reduced tendency to process information in its larger context coherence (Frith 1989), explicit instruction to attend to the semantic relationships between stimuli may overcome this tendency (Koldewyn et al. 2013). Therefore the use of an explicit task may have introduced compensatory strategies in ASD adults, allowing them to overcome their natural tendencies and leading to successful semantic integration in both modalities.

If this is the case, then an *implicit* semantic integration task, in which attention is not overtly drawn to the semantic relationships between stimuli, may be more likely to reveal a language-specific deficit in individuals with ASD. We explore this possibility in the current study, which aims to replicate our previous work (Coderre et al. 2017) with an implicit semantic priming task to determine whether semantic processing difficulties are observed when attention is not drawn to the semantic relatedness of stimuli. Adults with and without ASD viewed words and pictures that were either semantically related or unrelated and were told to monitor for “catch” trials (a string of consonants or a smiley face). That is, participants viewed pairs of related and unrelated stimuli but were not explicitly told about or instructed to attend to the relatedness of the stimuli. In this way, this type of paradigm should pick up on more implicit, “automatic” semantic processing. This type of implicit paradigm has been used in work with children with and without ASD (McCleery et al. 2010) and has previously shown to elicit N400 effects for TD participants in the absence of an explicit task. Therefore we expected that it would provide a sensitive measure of the degree to which semantic integration was triggered in the absence of overt instruction.

We predicted that both groups would show similar N400 effects for sematic processing of pictures, replicating previous findings that semantic processing of non-linguistic stimuli is intact in individuals with ASD. In contrast, if individuals with ASD have a reduced tendency to automatically and implicitly process the semantic properties of language, we would expect reduced N400 responses to words compared to TD individuals.

## Methods

### Participants

One group consisted of 20 adults with ASD, ages 18–54 (*M* = 27.8, *SD* = 9.5). The clinical diagnosis of autism or ASD (according to the DSM-IV or DSM-5, depending on the recency of the diagnosis/evaluation) was confirmed using the Autism Diagnostic Observation Schedule Second Edition (ADOS-2) (Lord et al. 2012). A second group consisted of 20 typically developing (TD) adults ages 19–49 (*M* = 25.2, *SD* = 7.57). The groups did not differ on age; however, the ASD group had significantly lower verbal and non-verbal IQ, as measured by the Kaufman Brief Intelligence Test Second Edition (KBIT-2; Kaufman and Kaufman 2004). There was also a trend toward lower receptive vocabulary knowledge in the ASD group, as measured by the Peabody Picture Vocabulary Test Fourth Edition (PPVT-4; Dunn and Dunn 2007). Finally, the groups also differed significantly on working memory, as assessed by a digit span task. All participants also completed the Autism Quotient (AQ), a self-report measure of autistic traits that is applicable for the general population (Baron-Cohen et al. 2001). As expected, the groups differed significantly on AQ scores, with higher scores in the TD group. Full participant demographics can be found in Table 1.

**Table 1:** Participant characteristics for the TD and ASD groups

	TD group (n = 20)	ASD group (n = 20)	Group difference
Age	25.2 (19–49)	27.8 (18–54)	*t*(36.3) = − 0.96, *p* = 0.34
PPVT	115 (98–135)	108 (80–132)	*t*(33.2) = 1.89, *p* = 0.067
K-BIT			
Verbal	118 (101–141)	107 (71–135)	*t*(33.3) = 2.54, *p* < 0.05
Non-verbal	112 (74–132)	101 (79–120)	*t*(37.5) = 2.38, *p* < 0.05
Autism Quotient	15.5 (5–37)	30.1 (17–43)	*t*(37.5) = -6.28, *p* < 0.001
Digit span	12 (7–16)	10 (4–16)	*t*(36.6) = 2.99, *p* < 0.01
WRAT^a^			
Word reading	111 (107–126)	108 (87–135)	*t*(18.4) = 0.70, *p* = 0.49
Sentence comprehension	119 (108–130)	104 (82–131)	*t*(16.8) = 2.95, *p* < 0.01
ADOS Module 4			
Social + communication total^b^	NA	9.3 (6–16)	NA
SA + RBB total^c^		12.3 (4–7)	
Calibrated severity score (CSS)^d^		6.6 (3–10)	

Means and ranges are reported for each measure. The ‘group difference’ column shows the results of independent-samples *t*-tests on each measure. Asterisks indicate statistically significant results (*p < 0.05; **p < 0.01; ***p < 0.001)

^a^Based on scores from 9/20 TD participants and 13/20 ASD participants

^b^The Social + communication total score is taken from the original algorithm for Module 4 of the ADOS-2, which is included in the scoring forms

^c^*SA* social affect, *RBB* restricted and repetitive behaviors. The SA + RBB total score is based off of a revised algorithm for Module 4 (Hus and Lord 2014), which was not included in the original release of the ADOS-2

^d^In generating Calibrated Severity Scores (CSS) for Module 4, only participants ages 9–39 were included by Hus and Lord (2014). No calibrated scores were provided for participants older than 40, which means that technically we cannot calculate CSS for our older participants. In our sample, three participants were over 40. When considering all participants under age 40 (n = 17), the average CSS was 6.9 (range 4–10)

All participants were able to read to an adequate degree to perform the word version of the task. All participants independently read the consent form and completed other questionnaires administered as part of this study and did not indicate any difficulty with understanding. We also obtained standardized measures of word reading and sentence comprehension (as assessed via the Wide Range Achievement Test, version 4; WRAT-4 Wilkinson and Robertson 2006) as part of a different study; although not all participants completed this assessment, the results from this task also confirmed that participants were able to read. As shown in Table 1, there were no differences in word reading scores between TD and ASD participants. The ASD group did show significantly lower scores on the sentence comprehension subtest compared to TD participants (*p* < 0.01), as would be expected based on the previously mentioned challenges with comprehension in this group. However, the scores for ASD participants for both word reading and sentence comprehension were all within the “normal” range (all above 70, i.e. within 2 standard deviations, SDs), confirming that their reading abilities were sufficiently advanced to read and understand the words presented as part of this paradigm.

All participants had normal or corrected-to-normal vision. Participants were recruited through newspaper advertisements, email announcements, and fliers at the University of Vermont and the Burlington, VT community. All procedures were approved by the University of Vermont Institutional Review Board. Written informed consent was obtained from all participants before testing. Participants were monetarily compensated for their participation.

### Stimuli and Procedure

The experimental paradigm used the same pairs of related and unrelated words and pictures as in our previous study (Coderre et al. 2017). Semantic relatedness was assessed using latent semantic analysis (LSA; mean LSA value for related pairs = 0.58; mean LSA value for unrelated pairs = 0.03). See Coderre et al. (2017) for more details regarding stimuli development.

**Fig. 1:**
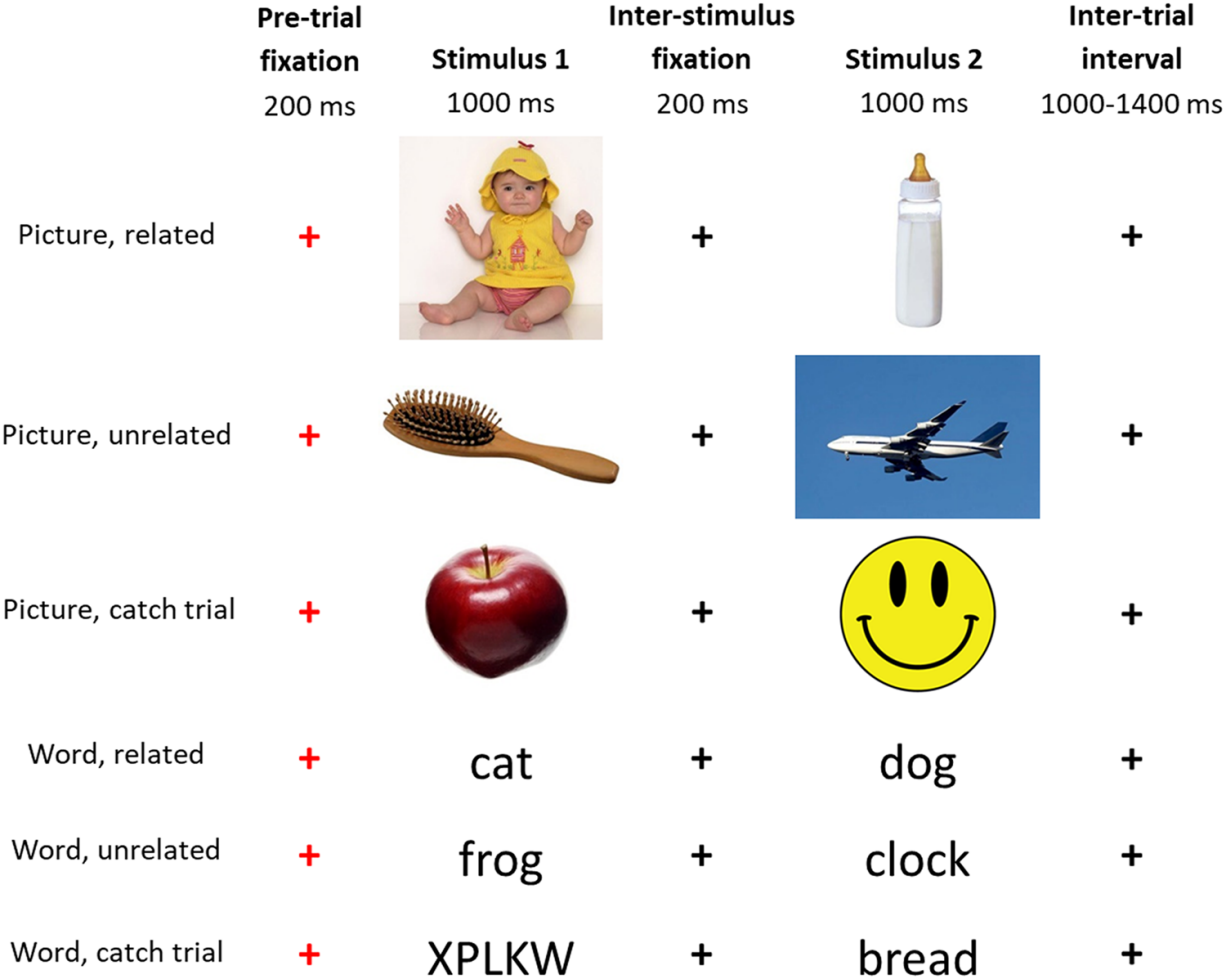
Example stimuli and timeline for the semantic priming task, in which participants viewed pairs of related or unrelated words and pictures. Catch stimuli (implicit task only) included a smiley face (picture conditions) or a consonant string in capital letters (word conditions)

To create an “implicit” paradigm, an additional 16% of trials (16 trials per block) were included as “catch trials” in which one stimulus was either a smiley face (for picture blocks) or a consonant string (for word blocks); see Fig. 1. Participants were asked to press a button on a button box whenever they saw a smiley face or a consonant string). Such catch trials have been used in prior research (McCleery et al. 2010) to ensure that participants attend to all stimuli. Note that although it includes a task, this paradigm assesses implicit semantic integration because participants are not asked to attend to the semantic relationship between the stimuli.

The implicit semantic priming task consisted of four blocks of picture pairs and four blocks of word pairs (25 related pairs, 25 unrelated pairs, 16 catch trials per block) for a total of 100 trials in each of the four stimuli types (picture related, picture unrelated, word related, word unrelated) plus 64 catch trials in each modality. Participants performed all four blocks of one modality followed by all four blocks of the other, counterbalanced across participants.

Stimuli were presented using E-Prime version 2.0.10.356. On each trial, a red fixation cross was presented for 400 ms, followed by the first word or picture for 1000 ms; an inter-stimulus blank screen for 300 ms; and the second word or picture for 1000 ms. Following the offset of the second stimulus a blank screen was presented for 400 ms, followed by a black fixation cross presented at an inter-trial interval ranging from 1000 to 1400 ms (mean 1200 ms).

Stimuli were presented on a Dell 21.5″ LCD monitor with a resolution of 1920 × 1080. Participants sat approximately 24″ away from the computer screen. Picture size ranged from 2.25 to 4″ in height and 2.5 to 4″ in width, yielding a visual angle between 5 and 9 degrees. Word stimuli were presented in size 28 Courier New font and ranged from 0.6 to 2.25″ in width and 0.25″ in height, yielding a visual angle between 1 and 5 degrees horizontally and 0.6 degrees vertically. During the semantic priming task, EEG data were recorded at 500 Hz using a 128-channel Geodesics Sensor net and NetStation version 5.3. Impedences were kept under 50 kΩ wherever possible. The entire experimental session lasted approximately 1.5 h including consenting, paperwork, EEG net application, and testing.

### Data Preprocessing

Data were preprocessed using EEGlab version 14.1.1 and Matlab 2017a. The data were filtered using a 0.1–50 Hz bandpass filter to remove very high- or low-frequency noise in the signal not related to brain activity. The data were then re-referenced using an average reference transform in order to express the voltage at each channel with respect to the average voltage across all channels (Dien 1998). The cleaned continuous data were then segmented into epochs time-locked to the onset of the second stimulus. Segments extended from 1400 ms before to 1000 ms after the second stimulus (in order to capture the response to the first stimulus, presented at -1300 ms). Eye movement artifacts were identified and removed from the data using independent component analysis (ICA). Prior to ICA decomposition, the mean of each trial was removed (Groppe et al. 2009) and the data were reduced to 32 dimensions. After ICA decomposition, eye movements, blinks, muscle artifacts, and other noise components were visually identified and manually removed from the data.

### Statistical Analyses

**Fig. 2:**
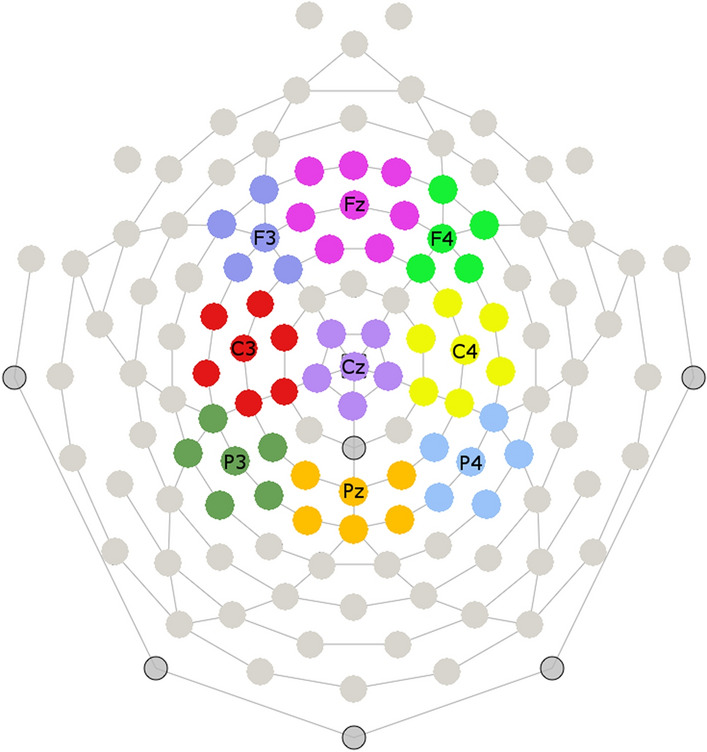
Illustration of the nine electrode clusters used for EEG analysis, with centers of each cluster labeled

ERP amplitude was evaluated at 9 regions across the scalp taken from the 10–20 distribution. Clusters were centered around F3, Fz, F4, C3, Cz, C4, P3, Pz, and P4 (Fig. 2). Since catch trials were relatively rare (16%) they likely elicited a P300 component that could confound N400 effects; therefore only experimental (i.e. non-catch) trials were included in analyses.

Because the N400 may occur at slightly different latencies between groups or modalities (e.g. Hamm et al. 2002; McPherson and Holcomb 1999; West and Holcomb 2002), we chose not to restrict our analyses to one predefined time window. We defined long time windows of interest based on visual inspection and then performed repeated-measure ANOVAs in 100 ms windows. ANOVAs were performed on the unrelated-related difference waves within each modality. *Group* (TD/ASD) was included as a between-subjects factor and *site* (frontal/central/parietal) and *laterality* (left/midline/right) were included as within-subjects factors. Because of group differences in the demographic variables, verbal and non-verbal K-BIT scores, PPVT scores, and digit span scores were included as covariates in all ANOVAs.

Although verbal and non-verbal IQ, receptive language scores, and digit span scores were included as covariates in the statistical analyses, there is the possibility that other demographic variables may have modulated ERP effects. Therefore in secondary analyses, we reran the ANOVAs described above with two modifications: using AQ score as a continuous variable instead of TD/ASD groups and also including age as a continuous variable. As can be seen in Table 1, our participants had quite a range of AQ scores, with notable overlap between the groups. One benefit of the AQ measure is that it can provide an estimate of autistic traits in the general population (Ruzich et al. 2015), since TD participants completed it as well. This assessment also provides a continuous measure of autistic traits, which allows us to explore finer-grained modulations of how autistic traits affect ERP responses compared to dichotomous grouping by the presence of an ASD diagnosis. In addition, as we have suggested elsewhere (Coderre et al. 2017), semantic integration abilities may also be mitigated by age; since we had a fairly wide range of ages in the current sample, we examined whether this variable might affect ERP responses, as well as how it might interact with AQ score. In summary, the ANOVAs for these secondary analyses were run on the unrelated-related difference waves in each modality and included *AQ score* and *age* as continuous variables, and *site* (frontal/central/parietal) and *laterality* (left/midline/right) as within-subjects factors, with interaction terms between all factors. Verbal and non-verbal K-BIT scores, PPVT scores, and digit span scores were included as covariates. These ANOVAs were run in the same analysis windows as specified above (100 ms windows from 200 to 800 ms). To interpret interactions between AQ score and age, scatterplots were created with regression lines for low-AQ and high-AQ participants (as determined by a median split: median AQ score = 21.5).

## Results

### Pictures

**Fig. 3:**
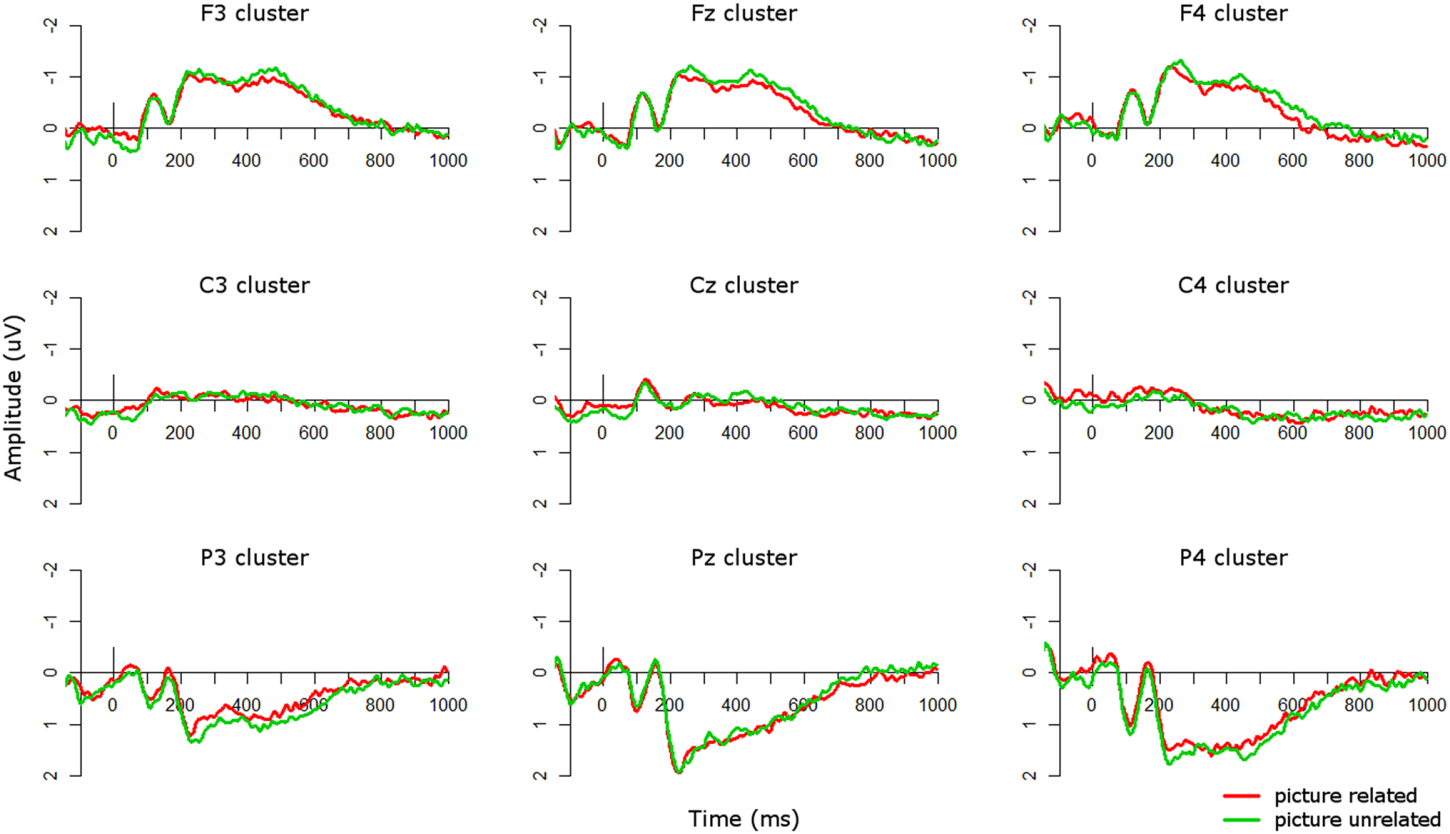
ERPs for picture conditions in the TD group. Negative is plotted upwards

**Fig. 4:**
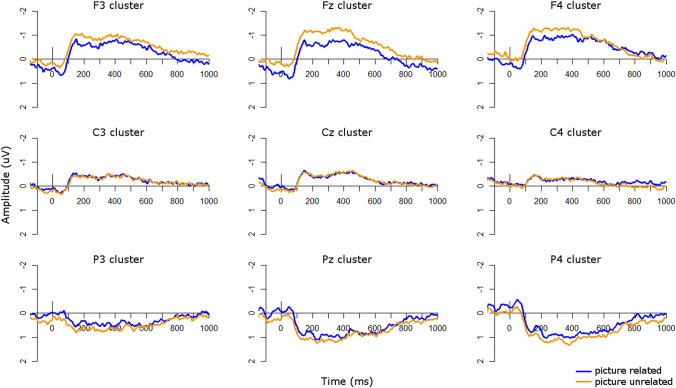
ERPs for picture conditions in the ASD group. Negative is plotted upwards

**Fig. 5:**
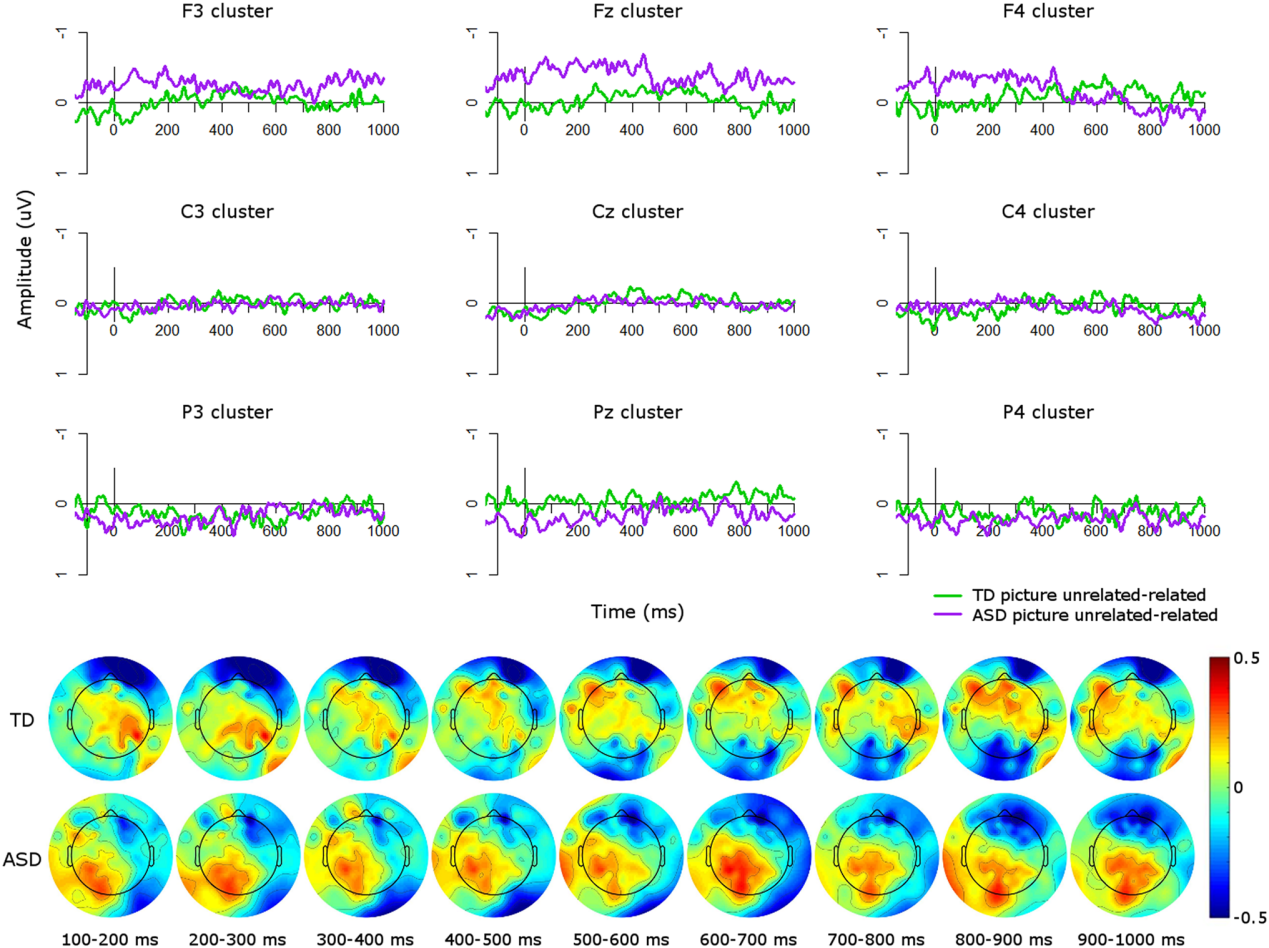
Group comparisons in the picture modality. Top: ERP difference waves (unrelated minus related) for each group in the picture modality; negative is plotted upwards. Bottom: Topographic plots of unrelated minus related conditions in the picture modality in 100 ms windows from 100 to 1000 ms for each group

Visual inspection of the ERP waveforms for the picture condition suggested that in the TD group (Fig. 3), the unrelated condition was more negative than the related condition over frontal sites from approximately 400–500 ms. This more frontal N400 effect is commonly seen for visual stimuli (Ganis et al. 1996; Hamm et al. 2002; McPherson and Holcomb 1999; Sitnikova et al. 2008), in contrast to the more centro-parietal scalp distribution of the N400 in response to linguistic stimuli (Kutas and Federmeier 2011). This effect was flipped over parietal sites, such that the unrelated condition was more positive than the related condition. The ASD group showed a similar pattern (Fig. 4), with unrelated conditions more negative than related conditions over frontal sites from approximately 200–400 ms over right frontal sites, with a more sustained effect from 200 to 1000 ms over left and midline frontal clusters. These effects can also be seen in Fig. 5, which plots the unrelated-related difference waves and topographic plots for each group.

To compare group effects in the picture condition, we ran repeated-measures ANOVAs in 100 ms time windows from 200 to 800 ms after presentation of the second stimulus. Full results are presented in Table 2; only significant (*p* < 0.05) main effects of group or interactions with group are discussed below.

**Table 2:** *F*-values for the repeated-measures ANOVAs on unrelated-related difference waves in each analysis window for the picture condition, with a between-subjects factor of *group* (TD, ASD) and within-subjects factors of *site* (frontal, central, parietal) and *laterality* (left, midline, right)

Main effect or interaction	200–300 ms	300–400 ms	400–500 ms	500–600 ms	600–700 ms	700–800 ms
PPVT	4.01	2.56	5.38 *	7.63 *	7.21 *	7.02 *
KBIT verbal	0.01	0.30	1.28	0.11	0.01	0.20
KBIT nonverbal	1.11	0.04	0.03	0.25	< 0.01	0.19
Digit span	0.55	0.76	0.56	1.58	0.58	0.92
Site	12.61 ***	8.31 ***	7.96 ***	5.03 **	4.83 *	1.12
Laterality	0.88	1.70	1.75	1.11	0.79	0.87
**Group**	6.59 *	2.22	2.33	1.43	1.96	1.36
**Group × site**	2.72	3.56 *	0.90	0.22	0.61	0.32
**Group × laterality**	0.15	0.17	0.21	1.03	0.58	2.07
Site × laterality	0.41	0.30	0.34	0.29	0.32	0.18
**Group × site × laterality**	1.30	1.67	1.01	0.99	2.30	3.07 *

Verbal and non-verbal K-BIT, PPVT, and digit span scores were included as covariates. Asterisks indicate statistically significant results (*p < 0.05; **p < 0.01; ***p < 0.001). Main effects of group and interactions with group are highlighted in bold

From 200 to 300 ms there was a significant main effect of group (*F*(1,34) = 6.59, *p* < 0.05) such that, over all electrode clusters, the ASD difference wave (*M* = − 0.07, *SD* = 0.55) was more negative than the TD difference wave (*M* = 0.04, *SD* = 0.51), indicating larger N400 effects in the ASD group than in the TD group (Fig. 5). To explore whether the relatedness effect was significant in either group individually, we also ran a repeated-measures ANOVA in each group with levels of *condition* (related/unrelated), *site* (frontal/central/parietal), and *laterality* (left/midline/right) in this time window. In the TD group there were no significant main effects of or interactions with condition (all *p*’s > 0.11). However, in the ASD group there was a significant interaction of condition and site (*F*(2,38) = 11.17, *p* < 0.001) such that over frontal sites the unrelated condition was more negative than the related condition, reflecting the more frontal N400 found for pictures (Ganis et al. 1996; Hamm et al. 2002; McPherson and Holcomb 1999; Sitnikova et al. 2008), whereas this effect was flipped over parietal sites, such that related was more negative than unrelated, reflecting the flipped dipole. Therefore the ASD group showed a significant N400 effect from 200 to 300 ms, particularly over frontal sites, but the TD group did not.

From 300 to 400 ms there was a significant interaction of group and site (*F*(2,76) = 3.56, *p* < 0.05). This arose from a significant effect of group at frontal sites (*F*(1,114) = 8.79, *p* < 0.01) such that the ASD difference wave (*M* = − 0.35, *SD* = 0.60) was more negative than the TD difference wave (*M* = − 0.07, *SD* = 0.52), indicating larger N400 effects in the ASD group than in the TD group (Fig. 5). To explore whether the relatedness effect was significant in either group individually, we also ran a repeated-measures ANOVA in each group with levels of *condition*, *site*, and *laterality* in this time window. In the TD group there were no significant main effects of or interactions with condition (all *p*’s > 0.32). However, in the ASD group there was a significant interaction of condition and site (*F*(2,38) = 8.54, *p* < 0.001) such that over frontal sites the unrelated condition was more negative than the related condition, whereas this effect was flipped at parietal sites. Therefore the ASD group showed a significant N400 effect over frontal sites from 300 to 400 ms. whereas the TD group did not.

From 700 to 800 ms there was a significant interaction of group, site, and laterality (*F*(4,152) = 3.07, *p* < 0.05) which arose from a trend of a main effect of group at frontal midline sites, i.e. the Fz cluster (*F*(1,34) = 3.01, *p* = 0.09). At this electrode cluster, the ASD difference wave (*M* = − 0.28, *SD* = 0.53) was more negative than the TD difference wave (*M* = − 0.02, *SD* = 0.47), indicating larger N400 effects in the ASD group than in the TD group. To explore whether the relatedness effect was significant in either group individually, we also ran a repeated-measures ANOVA in each group with levels of *condition*, *site*, and *laterality* in this time window. In the TD group there were no main effects of or interactions with condition (all *p*’s > 0.21). However, in the ASD group there was a significant interaction of condition and laterality (*F*(2,38) = 3.36, *p* < 0.05), which arose from trends of an effect of condition at midline (*F*(1,19) = 3.25, *p* = 0.09) and right hemisphere sites (*F*(1,19) = 3.94, *p* = 0.06). Over midline clusters the unrelated condition was more negative (*M* = 0.04, *SD* = 0.95) than the related condition (*M* = 0.11, *SD* = 0.84) whereas this effect was flipped over right hemisphere clusters, in which the related condition (*M* = − 0.06, *SD* = 0.88) was more negative than the related condition (*M* = 0.05, *SD* = 0.88. Therefore the ASD group showed a significant relatedness effect at midline electrode clusters, particularly at frontal sites, but a flipped effect over right-hemisphere clusters, whereas the TD group did not show any modulations of amplitude by relatedness.

As described above (see Statistical Analyses section), we also ran secondary analyses exploring how degree of autistic traits and age, and their potential interactions, might affect the results. To do so we ran repeated-measures ANOVAs on the unrelated-related difference waves in each of the specified analysis windows; AQ score and age were included as continuous variables, site and laterality were included as within-subjects variables, and verbal and non-verbal K-BIT scores, PPVT scores, and digit span scores were included as covariates. Visualization scatterplots were created to interpret interactions with age and AQ. The full results can be found in Table 3; only significant (*p* < 0.05) main effects of and interactions with AQ and age are discussed below.

**Table 3:** *F*-values for the repeated-measures ANOVAs on unrelated-related difference waves in each analysis window for the picture condition, with AQ score and age as continuous variables and within-subjects factors of *site* (frontal, central, parietal) and *laterality* (left, midline, right)

Main effect or interaction	200–300 ms	300–400 ms	400–500 ms	500–600 ms	600–700 ms	700–800 ms
PPVT	3.73	2.46	5.21 *	8.25 **	7.49 *	7.44 *
KBIT verbal	0.01	0.29	1.24	0.12	0.01	0.21
KBIT nonverbal	1.03	0.04	0.03	0.28	< 0.01	0.20
Digit span	0.51	0.73	0.54	1.71	0.60	0.97
**Age**	0.54	0.50	0.46	0.77	0.20	< 0.01
**AQ**	0.22	0.21	0.40	1.21	0.44	0.27
**Age × AQ**	5.03 *	2.10	2.33	4.36 *	4.76 *	5.15 *
Site	11.61 ***	7.47 ***	7.52 **	4.92 **	4.63 *	1.19
**Age × site**	1.11	1.01	0.62	1.35	0.51	4.12 *
**AQ × site**	0.20	0.05	0.04	0.03	< 0.01	0.13
**Age × AQ × site**	0.18	0.31	0.09	0.01	0.51	0.22
Laterality	0.94	1.76	1.77	1.06	0.74	0.83
**Age × laterality**	4.12 *	2.60	2.05	0.07	0.20	0.61
**AQ × laterality**	0.16	0.07	0.35	0.13	0.04	1.33
**Age × AQ × laterality**	0.54	1.03	0.13	1.08	0.25	0.66
Site × laterality	0.41	0.30	0.34	0.29	0.31	0.19
**Age × site × laterality**	1.07	0.78	0.45	0.26	0.27	0.33
**AQ × site × laterality**	0.77	0.93	0.94	1.25	1.80	2.51 *
**Age × AQ × site × laterality**	0.88	1.02	1.68	2.19	1.43	2.67 *

Verbal and non-verbal K-BIT, PPVT, and digit span scores were included as covariates. Asterisks indicate statistically significant results (*p < 0.05; **p < 0.01; ***p < 0.001). Main effects of and interactions with AQ and age are highlighted in bold

From 200 to 300 ms there was an interaction of age and laterality (*F*(2,72) = 4.12, *p* < 0.05). However, on follow-up, there were no main effects of AQ at any laterality.

From 200 to 300 ms, 500–600 ms, and 600–700 ms, there were interactions of age and AQ (all *p*’s < 0.05). In all time windows, visualization scatterplots indicated that increasing age was associated with larger N400 effects (such that unrelated conditions were more negative than related conditions) in participants with lower AQ scores, but the opposite trend (such that increasing age was associated with smaller N400 effects) happened in those with high AQ scores.

**Fig. 6:**
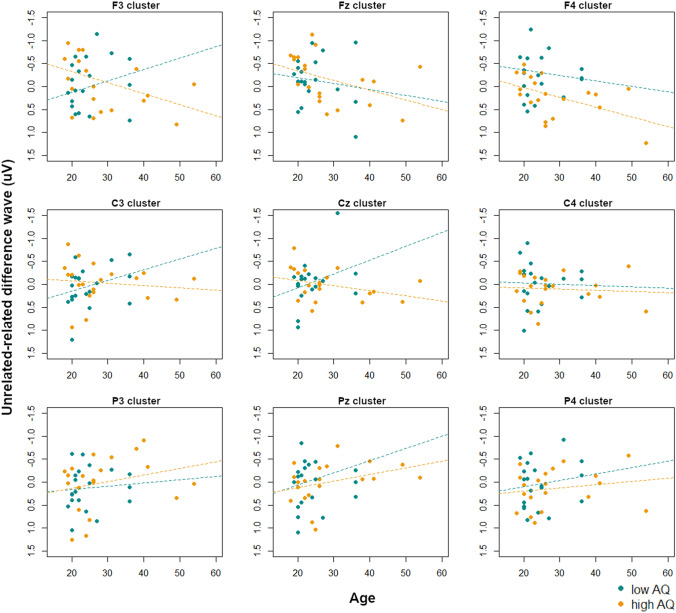
Interactions between age and AQ score on the N400 effect magnitude (unrelated-related difference waves) at each electrode cluster in the picture condition from 700 to 800 ms. Low-AQ and high-AQ participants (as determined by a median split) are indicated, with regression lines for each group. Negative is plotted upwards

From 700 to 800 ms there was an interaction of age, AQ, site, and hemisphere (*F*(4,144) = 2.66, *p* < 0.05), arising from an interaction of age and AQ at left and midline central sites, i.e. the C3 (*F*(1,32) = 4.77, *p* < 0.05) and Cz clusters (*F*(1,32) = 7.20, *p* < 0.05). Visualization scatterplots at each electrode cluster (Fig. 6) showed that at both sites, increasing age was associated with larger N400 effects (such that unrelated conditions were more negative than related conditions) in the low AQ group. This effect was flipped in high AQ participants, such that increasing age was associated with smaller N400 effects.

To summarize the findings in the picture modality, when grouping by TD/ASD designations the ASD group showed a significantly larger N400 effect (i.e. a more negative unrelated-related difference wave) in response to pictures than the TD group, particularly over frontal sites, from 200 to 400 ms and from 700 to 800 ms. When including AQ score (as a measure of autistic traits in the general population) and age as continuous variables in the analysis models, we also found complex interactions between these factors and the magnitude of the N400 effect. At both early (i.e. 200–300 ms) and late (500–800 ms) time windows, those with lower levels of autistic traits (i.e. lower AQ scores) showed increasing sensitivity to semantic relatedness of picture stimuli (i.e. larger N400 effect magnitude) with increasing age, whereas those with higher levels of autistic traits showed decreasing sensitivity to semantic relatedness. In most time windows, this effect was generalized over all electrode sites; from 700 to 800 ms, this effect was most prominent over left and midline central electrodes.

### Words

**Fig. 7:**
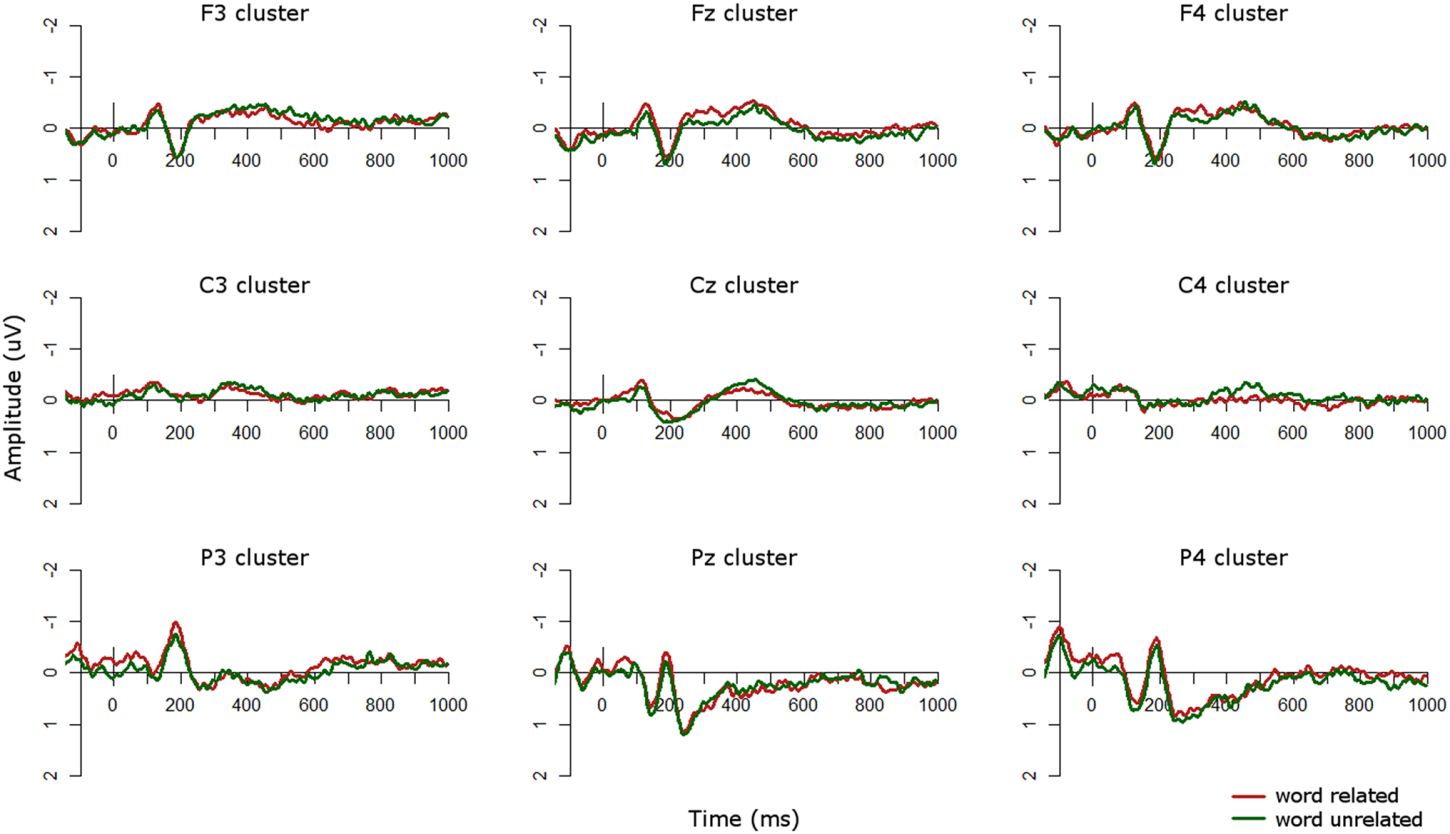
ERPs for word conditions in the TD group. Negative is plotted upwards

**Fig. 8:**
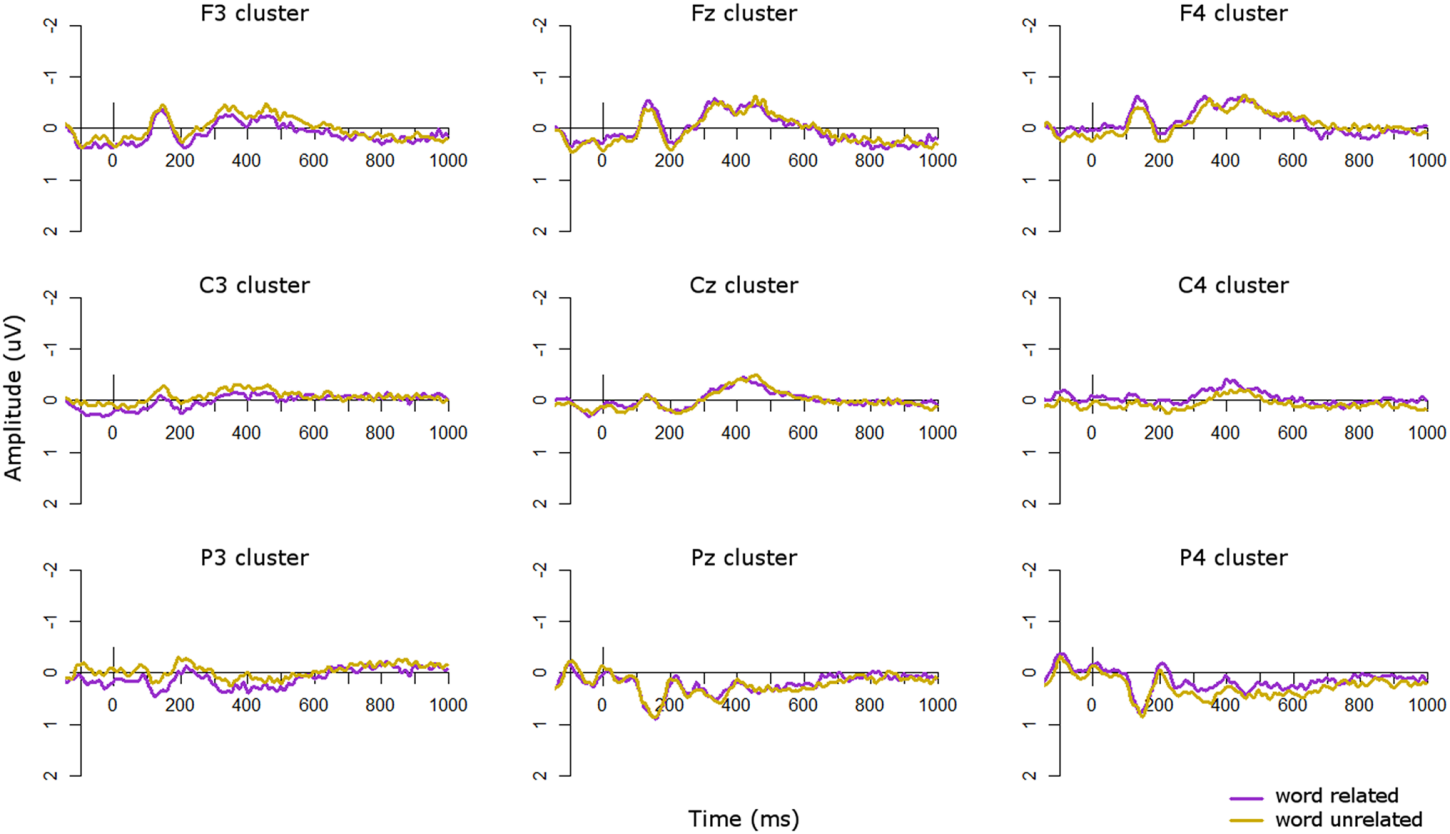
ERPs for word conditions in the ASD group. Negative is plotted upwards

Visual inspection of the ERP waveforms for the word condition suggested that in the TD group (Fig. 7) the unrelated condition was more negative than the related condition at central and right-hemisphere central sites from approximately 400–500 ms and at left frontal sites from approximately 500–700 ms. There was also an opposite effect, such that the unrelated condition was more positive than the related condition, at left parietal sites from approximately 500–600 ms and at right parietal sites from approximately 850–950 ms. In the ASD group (Fig. 8), the unrelated condition was more negative than the related condition at left-hemisphere sites, particularly over frontal scalp, from approximately 300–600 ms. This effect was flipped in the right hemisphere, particularly at right parietal sites, from approximately 300–500 ms.

**Fig. 9:**
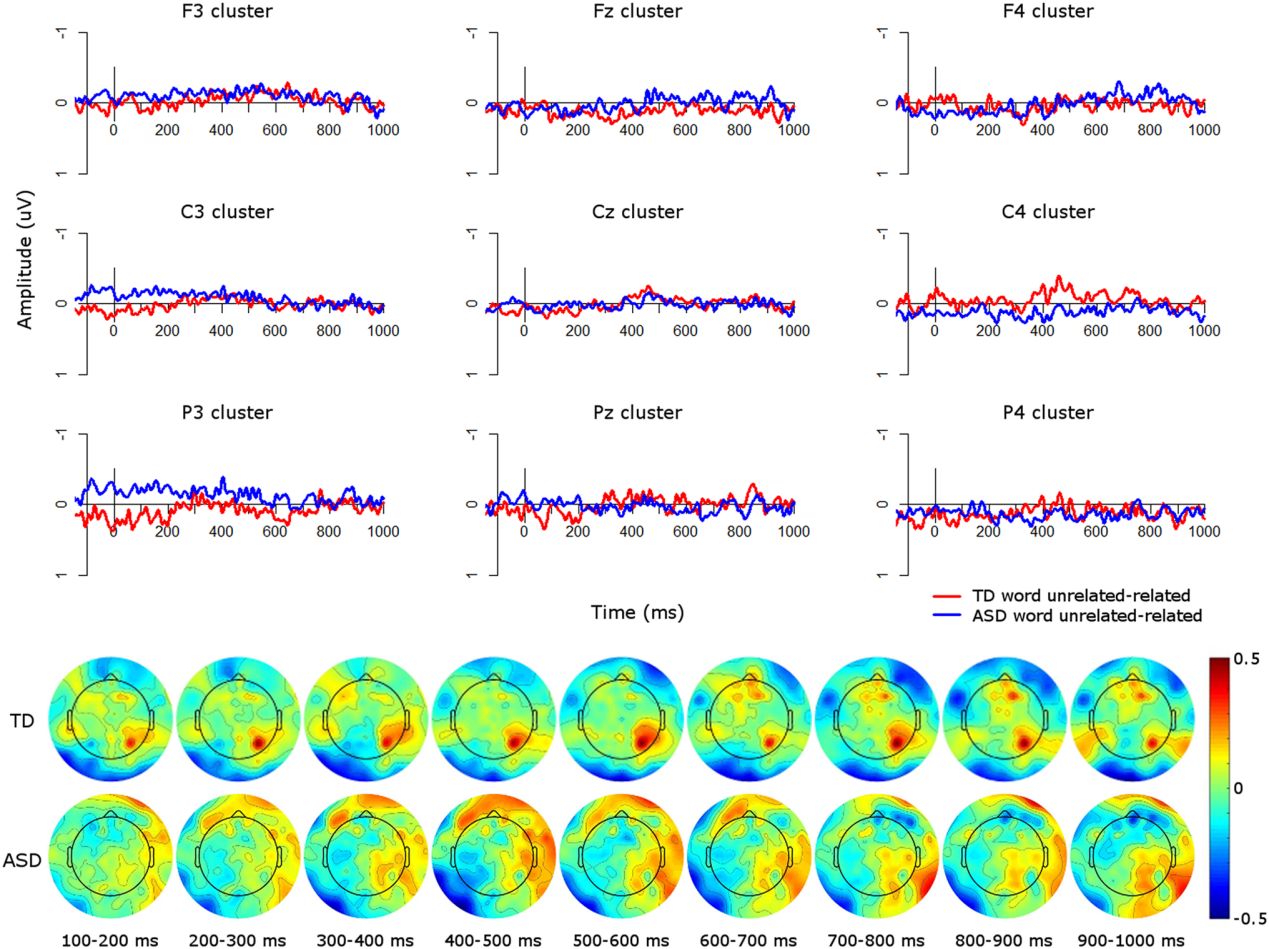
Group comparisons in the word modality. Top: ERP difference waves (unrelated minus related) for each group in the word modality; negative is plotted upwards. Bottom: Topographic plots of unrelated minus related conditions in the word modality in 100 ms windows from 100 to 1000 ms for each group

To compare group effects in the word condition, we ran repeated-measures ANOVAs in 100 ms time windows from 200 to 800 ms after presentation of the second stimulus (Table 4). However, there were no significant main effects of group or interactions with group in any time windows. This suggests that in the word modality there were no significant differences in N400 effects between the groups. This effect can also be seen in Fig. 9, which plots the unrelated-related difference waves and topographic plots for each group.

**Table 4:** *F*-values for the repeated-measures ANOVAs on unrelated-related difference waves in each analysis window for the word condition, with a between-subjects factor of *group* (TD, ASD) and within-subjects factors of *site* (frontal, central, parietal) and *laterality* (left, midline, right)

Main effect or interaction	200–300 ms	300–400 ms	400–500 ms	500–600 ms	600–700 ms	700–800 ms
PPVT	< 0.01	4.63 *	1.94	0.13	4.19 *	3.92
KBIT verbal	0.03	0.08	0.38	0.80	1.71	1.49
KBIT nonverbal	0.06	0.06	0.02	0.76	0.07	1.07
Digit span	2.99	0.03	< 0.01	< 0.01	0.73	0.27
Site	0.08	0.33	0.26	0.29	0.86	0.02
Laterality	5.03 **	6.41 **	2.24	1.47	0.26	0.29
**Group**	0.04	0.28	0.02	< 0.01	0.87	0.61
**Group × site**	0.14	0.86	0.33	0.39	0.36	0.41
**Group × laterality**	1.37	1.43	1.90	0.44	0.36	0.01
Site × laterality	0.90	1.09	1.41	1.41	0.99	1.42
**Group × site × laterality**	0.41	1.10	1.07	1.35	1.37	0.79

Verbal and non-verbal K-BIT, PPVT, and digit span scores were included as covariates. Asterisks indicate statistically significant results (*p < 0.05; **p < 0.01; ***p < 0.001). Main effects of group and interactions with group are highlighted in bold

To explore whether the unrelated-related condition effects were significant in either group individually, we also ran repeated-measures ANOVAs in each group with levels of *condition* (related/unrelated), *site* (frontal/central/parietal), and *laterality* (left/midline/right) in each time window. In the TD group there were no significant main effects of or interactions with condition in any time window. In the ASD group there were significant interactions of condition by hemisphere from 300 to 400 and 400 to 500 ms (all *p*’s < 0.05), which arose from a significant N400 effect (unrelated more negative than related) at left hemisphere sites from 300 to 500, with this effect flipped over right hemisphere sites from 300 to 400 ms. Therefore the ASD group showed an N400 effect in response to words from 300 to 500 ms but the TD group did not, although the groups did not differ significantly during direct comparisons.

As described above, we also ran secondary analyses exploring how autistic traits and age, and their potential interactions, might affect results. To do so we ran repeated-measures ANOVAs on the unrelated-related difference waves in each of the specified analysis windows; AQ score and age were included as continuous variables, site and laterality were included as within-subjects variables, and verbal and non-verbal K-BIT scores, PPVT scores, and digit span scores were included as covariates. The full results can be found in Table 5; only significant (p < 0.05) main effects of and interactions with AQ and age are discussed below.

**Table 5:** *F*-values for the repeated-measures ANOVAs on unrelated-related difference waves in each analysis window for the word condition, with AQ score and age as continuous variables and within-subjects factors of *site* (frontal, central, parietal) and *laterality* (left, midline, right)

Main effect or interaction	200–300 ms	300–400 ms	400–500 ms	500–600 ms	600–700 ms	700–800 ms
PPVT	< 0.01	4.50 *	1.88	0.13	4.06	3.86
KBIT verbal	0.03	0.08	0.37	0.78	1.67	1.47
KBIT nonverbal	0.05	0.06	0.01	0.74	0.07	1.05
Digit span	2.85	0.03	< 0.01	< 0.01	0.71	0.27
**Age**	0.09	0.50	0.16	0.86	0.69	0.86
**AQ**	0.31	0.23	0.80	0.06	< 0.01	0.04
**Age × AQ**	< 0.01	0.54	0.01	0.03	1.07	1.25
Site	0.08	0.34	0.26	0.29	0.85	0.02
**Age × site**	2.49	0.97	0.07	0.84	1.03	1.30
**AQ × site**	0.15	0.30	0.38	0.97	0.33	0.11
**Age × AQ × site**	0.16	1.79	2.80	0.16	0.33	0.06
Laterality	4.77 *	6.20 **	2.14	1.44	0.25	0.28
**Age × laterality**	0.02	0.53	0.42	1.21	0.79	0.57
**AQ × laterality**	0.45	1.40	1.65	0.02	0.01	0.02
**Age × AQ × laterality**	0.82	0.19	0.07	0.41	0.02	0.01
Site × laterality	0.96	1.16	1.53	1.51	1.09	1.48
**Age × site × laterality**	1.75	1.83	1.64	2.19	1.11	1.06
**AQ × site × laterality**	1.26	1.33	1.21	1.93	2.71 *	0.71
**Age × AQ × site × laterality**	1.87	2.53 *	3.57 **	2.18	3.54 **	2.76 *

Verbal and non-verbal K-BIT, PPVT, and digit span scores were included as covariates. Asterisks indicate statistically significant results (*p < 0.05; **p < 0.01; ***p < 0.001). Main effects of and interactions with AQ and age are highlighted in bold

From 300 to 500 and 600 to 800 there were significant interactions of age, AQ, site, and laterality.

From 300 to 400 ms, this interaction (*F*(4,144) = 2.53, *p* < 0.05) arose from an interaction of age, AQ, and site at midline lateralities (*F*(2,72) = 3.32, *p* < 0.05). However, on follow-up, there were no interactions of age and AQ at any site (all *p*’s > 0.20).

**Fig. 10:**
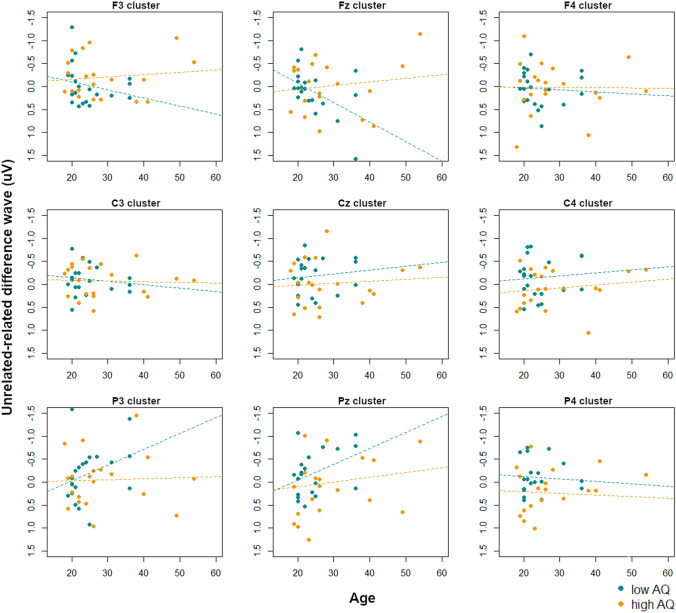
Interactions between age and AQ score on the N400 effect magnitude (unrelated-related difference waves) at each electrode cluster in the word condition from 400 to 500 ms. Low-AQ and high-AQ participants (as determined by a median split) are indicated, with regression lines for each group. Negative is plotted upwards

From 400 to 500 ms, the interaction of age, AQ, site, and laterality (*F*(4,144) = 3.57, *p* < 0.01) arose from an interaction of age and AQ at left and midline clusters (all *p*’s < 0.05). In the left hemisphere, follow-up tests showed no interactions of age and AQ at any site (all *p*’s > 0.20). At midline lateralities, there was a trend of an interaction of age and AQ at frontal sites (*F*(1,32) = 3.45, *p* = 0.07), i.e. the Fz cluster. Visualization scatterplots (Fig. 10) showed that at the Fz cluster, participants with lower AQ scores showed decreasing N400 effects (such that unrelated conditions were less negative than related conditions) with increasing age, whereas this was not as strong as for those with higher AQ scores. However, this effect was flipped over parietal sites, particularly the P3 and Pz clusters, such that those with lower AQ scores showed increasing N400 effects with age whereas this relationship was less pronounced for those with higher AQ scores. Because the N400 effect to linguistic stimuli tends to be more centro-parietal (Kutas and Federmeier 2011), and we indeed observed an N400 effect at central sites in the TD group (although not statistically significant), we interpret this parietal relationship as the “typical” N400 effect. Therefore age and AQ seem to interact in similar ways as in the picture condition, with increasing age associated with larger N400 effects (though with different scalp distributions reflecting modality differences in the N400 effect) in those with lower AQ scores, whereas this effect is not as strong in those with higher AQ scores.

**Fig. 11:**
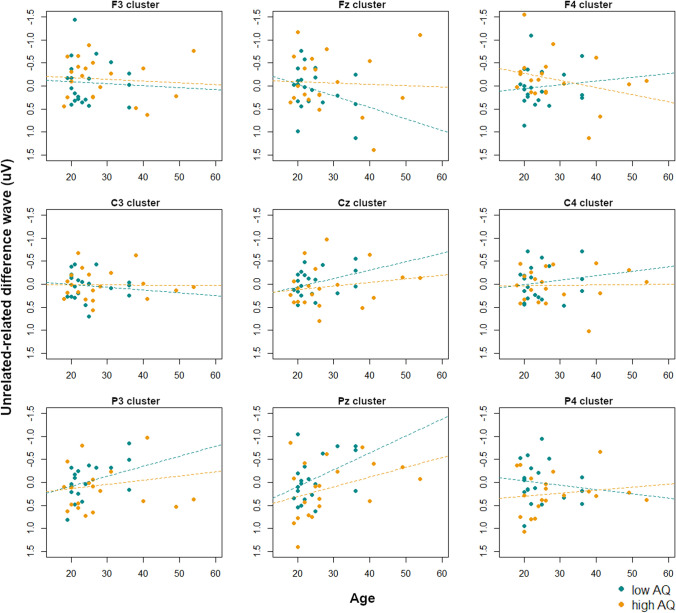
Interactions between age and AQ score on the N400 effect magnitude (unrelated-related difference waves) at each electrode cluster in the word condition from 600 to 700 ms. Low-AQ and high-AQ participants (as determined by a median split) are indicated, with regression lines for each group. Negative is plotted upwards

From 600 to 700 ms, the interaction of age, AQ, site, and laterality (*F*(4,144) = 3.54, *p* < 0.01) arose from an interaction of age and AQ at right frontal sites, i.e. the F4 cluster (*F*(1,32) = 3.29, *p* = 0.08). Visualization scatterplots (Fig. 11) showed that at the F4 cluster, participants with lower AQ scores showed increasing N400 effects (such that unrelated conditions were more negative than related conditions) with increasing age, whereas the opposite pattern occurred for those with higher AQ scores. Over most central and parietal clusters, the high and low AQ participants showed similar patterns such that increasing age was associated with larger N400 effects, although this effect was not as strong in the participants with higher AQ scores.

From 700 to 800 ms, there was an interaction of age, AQ, site, and laterality (*F*(4,144) = 2.76, *p* < 0.05). However, on follow-up testing there were no significant interactions of age and AQ at any site or laterality.

To summarize the findings in the word modality, when grouping by TD/ASD designations we observed no significant group differences in N400 magnitude. This is somewhat surprising and contradicts previous literature showing that individuals with ASD experience reduced or absent N400 effects to linguistic stimuli. However, when including AQ score and age in the analysis models, we found significant interactions between these variables in the N400 window (400–500 ms). Specifically, in participants with less severe autistic symptoms (i.e. lower AQ scores), sensitivity to semantic relatedness (i.e. N400 effect magnitude) increased with age over centro-parietal sites (reflecting the typical N400 scalp distribution in response to linguistic stimuli; Kutas and Federmeier 2011). This effect was flipped over frontal sites, such that lower AQ scores were associated with decreasing N400 magnitude with age, likely reflecting the flipped dipole of the N400. However, in participants with higher levels of autistic traits, this sensitivity to semantic relatedness was not as strongly modulated by age and did not show as much topographic variation, with fairly similar effects found over all sites. A similar pattern was also observed in a slightly later window from 600 to 700 ms, with the most significant effects coming out over right frontal electrodes such that in participants with lower levels of autistic traits, increasing age was associated with larger N400 effects, whereas this relationship was flipped over centro-parietal sites.

## Discussion

This study built on previous work by our group (Coderre et al. 2017) by examining implicit semantic integration in visual and linguistic modalities in individuals with ASD. Adults with and without ASD viewed words and pictures that were either semantically related or unrelated during concurrent EEG recording. Our primary ERP component of interest was the N400 component, an index of semantic processing. We predicted similar N400 effects between groups for sematic processing of pictures, which would support previous findings that semantic processing of non-linguistic stimuli is intact in individuals with ASD. In contrast, we predicted smaller N400 effects for the ASD group for semantic processing of words, which would suggest a reduced tendency to automatically and implicitly process the semantic properties of language in this population.

In the picture condition, both groups appeared to show sustained N400 effects over frontal sites. This longer and more frontally-distributed N400 effect is characteristic of visual stimuli (Coderre et al. 2017, 2018; Cohn et al. 2012; West and Holcomb 2002), so is in line with previous literature. In group comparisons, the ASD group showed significantly larger N400 effects than the TD group, particularly over frontal sites and between 200–400 ms. Although we had predicted that the groups would not differ on the magnitude of their N400 effects for visual stimuli, suggesting intact semantic processing for non-linguistic information as has been reported previously (Coderre et al. 2017; Kamio and Toichi 2000; McCleery et al. 2010), this result of larger N400 effects for the ASD group actually suggests that visuo-semantic processing may be *enhanced* in individuals with ASD compared to TD individuals. This interpretation would be in line with previous studies suggesting that lower-level perceptual processing is enhanced in individuals with ASD (O’Riordan et al. 2001; O’Riordan and Plaisted 2001) and would fit in with claims in the educational literature that visual supports can be useful in improving reading comprehension in individuals with ASD (Gately 2008; Nguyen et al. 2015; Styslinger 2012). However, we offer this interpretation with the caveat that our potential finding of enhanced visuo-semantic processing in individuals with ASD appears in response to single pictures; comprehension of more complex stimuli like narratives may still be difficult for individuals with ASD, even in visual modalities (Coderre 2020; Coderre et al. 2018).

In the word condition, both groups appeared to show small N400 effects, from 400 to 500 ms over central scalp in the TD group and from 300 to 600 ms over frontal scalp in the ASD group. However, the groups did not differ on the magnitude of their N400 effects, as evidenced by the lack of significant group effects in the overall ANOVAs. Although at first glance this appears to support our previous finding (Coderre et al. 2017) that there were no differences in the magnitude of the N400 effect between ASD and TD groups, upon additional examination in each group individually we actually found the opposite effect as predicted: the ASD group showed a statistically significant N400 effect over left-hemisphere sites from 300 to 500 ms, but the TD group did not show any significant effects of relatedness.

The fact that our TD group did not show an N400 effect in the word modality is curious. One possibility for this finding is that the implicit task we employed here may be very subtle, and not strong enough to elicit reliable N400 effects. For example, visual inspection of the ERP waveforms in Fig. 7 suggested that a small N400 was present, although not statistically significant. However, this type of implicit paradigm has been used in other studies previously (McCleery et al. 2010) and is not dissimilar from many other semantic priming paradigms that do not require overt attention to semantic relatedness. For instance, many semantic priming paradigms require participants to make a lexical decision on the target word and present the prime so fast that it is considered “unconscious” semantic priming (McNamara 2005; Neely 1991). Another possibility may be that, given the sparsity of required responses, participants were not attending to every stimulus. However, behavioral performance on the catch trials was high (average hit rate = 97%), indicating that participants were monitoring the stimuli appropriately and attending to the task. Additional insights may come from our secondary analyses investigating autistic traits as a continuous measure.

### Effects of AQ and Age

One possibility for the lack of significant group differences in the word condition when splitting by TD/ASD designation is that we were not adequately capturing the full spectrum of variation in autistic traits. In secondary analyses, we included AQ score (a self-report measure of autistic traits in the general population) in our model, as well as possible interactions of AQ score and age. Although we originally classified our participants according to the presence of an ASD diagnosis, our participant sample included a fairly wide range of AQ scores that spanned diagnostic groups. The continuous nature of this measure (with higher scores indicating higher degrees of autistic traits) also allowed for finer-grained explorations of how autistic traits might affect N400 amplitudes. Age was also included because of the fairly wide age range in our sample and because we had previously postulated that semantic integration abilities may also be mitigated by age, particularly in ASD participants (Coderre et al. 2017). Inclusion of these variables in our analyses revealed complex interactions of age and level of autistic traits that modulated N400 effects in interesting ways.

In the word condition, we observed interactions of age and AQ score in the N400 window (300–500 ms) such that in participants with less severe autistic symptoms (i.e. lower AQ scores), sensitivity to semantic relatedness (i.e. N400 effect magnitude over centro-parietal sites) increased with age. Although the N400 ERP component has been found to get smaller and shift its peak later in older age (Kutas and Iragui 1998), its magnitude is typically greater in adulthood than in early adolescence (Benau et al. 2011), which fits with the age range in our participant sample and the findings in our data. Interestingly, we also observed that although increasing age was associated with greater N400 amplitude, in participants with higher levels of autistic traits (i.e. higher AQ scores) this sensitivity to semantic relatedness was not as strongly modulated by age.

These interactions with age in the word condition, such that younger ages were associated with smaller N400 effects (in participants with both high and low AQ scores, although this effect was stronger in low AQ participants), fits in with an interpretation offered by Coderre et al. (2017), who proposed that individuals with ASD develop compensatory mechanisms as they grow older to make up for their difficulties with semantic processing of language. By this interpretation, Coderre et al. also suggested that larger impairments in semantic processing should be observable in children compared to adults with ASD, since children may have underdeveloped compensatory mechanisms. The relationship observed here between age and N400 effect suggests the same interpretation: that younger age should be associated with smaller N400 effects to language in participants with ASD.

In the picture condition, we observed interactions of age and AQ score at both early (i.e. 200–300 ms) and late (i.e. 500–800 ms) time windows, such that, similar to the pattern observed in the word condition, lower levels of autistic traits (i.e. lower AQ scores) were associated with increasing sensitivity to semantic relatedness of picture stimuli (i.e. larger N400 effect magnitude) with increasing age. In those with higher levels of autistic traits, however, an opposite effect was found between picture and word conditions: for pictures, increasing age was associated with *smaller* N400 effects, whereas for words, increasing age was associated with *larger* N400 effects. This could suggest a trade-off effect: that as individuals with more severe autistic symptoms get older, evaluating semantic relatedness gets easier for words but harder for pictures. If, as we have proposed, individuals who struggle with semantic integration develop compensatory strategies over the course of their development, the increased attention and cognitive demands required by such mechanisms may lead to detrimental effects on other aspects of semantic processing, such as in the visual domain. This trade-off effect may be explained by the historic importance of foundational literacy (e.g., reading, writing, and mathematics) in the education system. Although recent trends have shown schools starting to expand students’ understanding of other types of literacy (e.g., social-emotional or health), and although emerging research is showing many similar cognitive processes underlying literacy in different modalities such as language and sequential images (e.g. Coderre et al. 2018; Cohn et al. 2012), foundational literacy has always been the key focus of education. If students are learning and practicing how to fluently decode, comprehend, and express their understanding of words, it would make sense that as adults it is an easier skill than understanding pictures. If this skill is practiced more frequently, it may explain why adults have an easier time understanding the stimuli presented in the word form and not the picture. While this interpretation is speculative, our findings of complex relationships between age, autistic traits, and semantic processing in picture and language modalities offers rich opportunities for future research.

### Additional Considerations

Interestingly, the fact that we observed similar patterns of interactions of age and AQ score for both word and picture conditions—such that age modulated N400 effects more strongly in those with lower AQ scores than those with higher AQ scores—suggests that higher levels of autistic traits may be associated with increased difficulty in semantic integration *regardless of modality*. This finding contradicts the proposal made by others in the literature suggesting that individuals with ASD have a language-specific deficit in semantic integration (Kamio and Toichi 2000; McCleery et al. 2010). In contrast, it suggests that semantic processing difficulties are characteristic of autistic traits, and that these difficulties also extend to non-linguistic modalities. This suggests a domain-general impairment in semantic integration, in line with conclusions made by other studies investigating higher-level comprehension abilities such as narrative processing (Coderre 2020; Coderre et al. 2018). Importantly, the fact that we were able to pick up this relationship between level of autistic traits and semantic processing even with relatively simplistic stimuli—pairs of single pictures and words—suggests that more severe symptoms may be associated with difficulties in integrating even these simple stimuli. In other words, whereas semantic processing difficulties may become most apparent in those with milder symptoms at the sentence or narrative comprehension level and not at the single-word level (in line with proposals of autism as a disorder of more complex information processing: Minshew et al. 1997; Minshew and Goldstein 1998), our findings of relationships between symptom severity and N400 amplitude suggests that individuals who are more severely affected may also have more trouble with simpler processing such as integrating the meanings of single words and pictures. Such difficulties at lower levels of semantic processing would no doubt be compounded at more challenging levels of comprehension such as sentences and narratives.

An additional important point to mention is the utility of the AQ in examining continuous measures of autistic traits across participants. Whereas the “gold standard” diagnostic measure of autism, the ADOS, can be used to obtain severity scores, it is typically only administered to participants in the predefined ASD group; due to the time it takes to administer, it is not often obtained for TD participants. However, the AQ is a useful measure because it is a fairly short self-report survey and can be used to assess autistic symptomology in the general population, including in TD participants (Ruzich et al. 2015). As demonstrated in our data, the AQ also provides a continuous measure of autistic symptomology which can elucidate complex and interesting modulations of results: insights which may not be possible when simply grouping participants into TD/ASD groups. Indeed, in the current data, our initial analyses comparing TD/ASD groups yielded limited insight into the nature of semantic processing of word stimuli, yet the inclusion of AQ score in analyses brought out many interesting findings. In our current conceptualization of autism as a spectrum disorder, with many individuals self-diagnosed (Lewis 2016), delineating participants according to the presence of a diagnosis may be losing its potency. For instance, a recent meta-analysis demonstrated that the effect sizes in psychology studies comparing ASD and TD populations have diminished in recent years (Rødgaard et al. 2019), likely because of changes to the diagnostic criteria and a blurring of the lines between what is “normal” and what is not. Our findings support this claim and suggest that, in the future of autism research, levels of autistic traits in the general population may be more significant in understanding language processing than whether an individual holds a diagnosis.

A final point to mention is the potential of implicit stimuli to investigate semantic processing in individuals across the autism spectrum, including in those with more limited language or verbal skills. One benefit to the current implicit paradigm is that differences in N400 effects can be observed even in the absence of overt behavioral responses. This potentially opens up investigations of semantic processing to extend beyond participants who are able to understand task directions and make overt behavioral responses to indicate their understanding of semantic relatedness, as we have explored in prior studies (Coderre et al. 2019). Because the current paradigm uses written words, even investigations of implicit processing would require participants to be able to read, restricting the sample population who can take part in this research. However, future investigations using a similar paradigm with auditory words could be used, which would expand the population of individuals with ASD who could participate. Overall, we believe that both ERPs and implicit paradigms hold great potential for exploring language processing in individuals across the entire autism spectrum, including those who have little-to-no functional language.

## Conclusions

In conclusion, the current study investigated semantic integration of word and picture stimuli using an implicit semantic priming task. We observed complex interactions between age and autistic traits, such that those with higher levels of autistic symptomatology demonstrated weaker modulations of the N400 ERP component with age. This trend was consistent across both picture and word modalities, suggesting a domain-general difficulty in semantic processing in those with higher levels of autistic traits. Our results point to the utility of using continuous measures of autistic traits rather than simply grouping by the presence of a diagnosis of ASD, which holds significant implications for how we conceptualize and compare groups in autism research and also may be potentially beneficial information for practitioners in the autism field. With recent changes to diagnostic criteria and increasingly more individuals self-diagnosing or foregoing formal diagnoses altogether, the conceptualization of autistic traits as being present to varying degrees in the general population serves to underscore the message of the modern neurodiversity movement: that these variations are normal, rather than deficits.
